# Metabolite Modulation in Human Plasma in the Early Phase of Acclimatization to Hypobaric Hypoxia

**DOI:** 10.1038/srep22589

**Published:** 2016-03-04

**Authors:** Wen-Ting Liao, Bao Liu, Jian Chen, Jian-Hua Cui, Yi-Xing Gao, Fu-Yu Liu, Gang Xu, Bing-Da Sun, Er-Long Zhang, Zhi-Bin Yuan, Gang Zhang, Yu-Qi Gao

**Affiliations:** 1 Institute of Medicine and Hygienic Equipment for High Altitude Region, College of High Altitude Military Medicine, Third Military Medical University, Chongqing 400038, China; 2 Department of Pathophysiology and High Altitude Physiology, College of High Altitude Military Medicine, Third Military Medical University, Chongqing 400038, China; 3 Department of High Altitude Military Hygiene, College of High Altitude Military Medicine, Third Military Medical University, Chongqing 400038, China; 4 Key Laboratory of High Altitude Medicine (Third Military Medical University), Ministry of Education, Chongqing 400038, China; 5 The Key Laboratory of High Altitude Medicine, People’s Liberation Army, Chongqing 400038, China; 6 18^th^ Hospital, People’s Liberation Army, Yecheng, Xinjiang, China

## Abstract

The exposure of healthy subjects to high altitude represents a model to explore the pathophysiology of diseases related to tissue hypoxia. We explored a plasma metabolomics approach to detect alterations induced by the exposure of subjects to high altitude. Plasma samples were collected from 60 subjects both on plain and at high altitude (5300 m). Metabolite profiling was performed by gas chromatography-mass spectrometry (GC-MS) and ultra-performance liquid chromatography-quadrupole time-of-flight mass spectrometry (UPLC-QTOFMS) in conjunction with univariate and multivariate statistical analyses. ELISA assays were further employed to measure the levels of several relevant enzymes together with perturbed metabolic pathways. The results showed that hypobaric hypoxia caused significant and comprehensive metabolic changes, as represented by significant changes of 44 metabolites and 4 relevant enzymes. Using MetaboAnalyst 3.0, it was found that several key metabolic pathways were acutely perturbed. In addition, 5 differentially expressed metabolites in pre-exposure samples from the acute mountain sickness-susceptible (AMS-S) group compared with those from the AMS-resistant (AMS-R) group are identified, which warrant further validation as potential predictive biomarkers for AMS-S individuals. These results provide new insights for further understanding the pathophysiological mechanism of early acclimatization to hypobaric hypoxia and other diseases correlated to tissue hypoxia.

Hypoxia is a pervasive physiological stimulus that is encountered under various cellular conditions, such as high altitude, physical exercise, pregnancy, aging, inflammation, cardiovascular and respiratory failures, wounds and even cancer. The study of the mechanisms whereby the human body adapts to hypoxia occurring as a consequence of hypobaric conditions defines the field of high altitude medicine and has implications for the pathophysiology of diseases correlated to tissue hypoxia, *e.g.* heart failure, severe obesity and obstructive sleep apnea syndrome.

With an increasing number of people moving to high altitude, the study of physiological acclimatization to hypoxia and related diseases is growing in importance. Identifying the molecular variables that play key roles in this process is important in elucidating the mechanisms known to counteract the negative effects of oxygen deficiency. The physiological processes characterizing adaptation to acute and prolonged hypobaric hypoxia exposure at high altitude include pulmonary, cardiac and hemeatological changes. The processes of adaptation to hypoxia are likely to reflect the modulation of related metabolites as previously demonstrated^1^.

Genomics and proteomics have merged as biochemical profiling tools to provide important insights into the biology of hypoxia-related conditions^1^. Although these profiling approaches focus on upstream genetic and protein variations, whereas the discipline of metabolomics captures the global metabolic changes that occur in response to pathological, environmental or lifestyle factors^2^. Consequently, metabolomics complements the information obtained by genomics and proteomics and has already shown promise in identifying metabolite-based biomarkers of acute and chronic hypoxia, including stroke^3^, cardiovascular diseases^4^ and various cancers^5^. Recently, nuclear magnetic resonance (NMR)-based metabolomics have enabled study of the effects of acute hypobaric hypoxia on metabolic profiles^6^,^7^,^8^,^9^,^10^. Metabolic profiles of animal models have been used to reveal changes in energy metabolism with the use of an anti-anxiety herb formula^6^ or with vitamin supplements^7^. Metabolic profiles of healthy human volunteers subjected to 8 h of 12% oxygen have shown changes in Hypoxia-Inducible Factor 1 HIF-1 levels and oxidative stress^8^. Our metabolomic study of high-altitude pulmonary edema using high resolution ^1^H NMR spectroscopy identified a panel of 20 differential plasma metabolites, including valine, leucine, citrate and glucose, demonstrating that metabolic profiles can be used in the discovery of biomarkers of disease^9^. Recently, Lou *et al*. (2014) employed urinary liquid chromatography-mass spectrometry (LC-MS)-based metabolomic analysis to study systemic changes resulting from acute hypoxia and found that purine metabolic products (uric acid, xanthine and hypoxathine) and 1-methyladenosine were highly upregulated after rapid exposure to a hypoxic environment^10^.

In this study, we conducted a comprehensive analysis of the plasma metabolites in 60 subjects both on plain and on the fourth day after arriving at high altitude (5300 m) using gas chromatography-mass spectrometry (GC-MS) and ultra-performance liquid chromatography-quadrupole time-of-flight mass spectrometry (UPLC-QTOFMS). The metabolic variations were comprehensively investigated and were cross-checked by the two analytical methods. Our results show the perturbation of metabolically-relevant pathways during early acclimatization to high altitude, including inflammatory response-related metabolism, energy metabolism, bile acid metabolism and heme metabolism. Five metabolites showed significantly higher or lower levels in pre-exposure samples from acute mountain sickness susceptible (AMS-S) subjects compared with those from acute mountain sickness resistant (AMS-R) subjects, and may be potential predictive biomarkers for AMS-S individuals. This work provides new insights into the pathophysiological changes of hypobaric hypoxia and the prediction of AMS-S individuals. A flow chart illustrating the study design is shown in Fig. 1.

## Results

### Clinical Characteristics of Subjects

The clinical characteristics of the subjects are in Table 1. It is well established that rapid ascent of subjects to a high altitude causes a cascade of physiological responses. However, none of the 60 subjects in this study progressed to the more severe and potentially fatal forms of high-altitude pulmonary edema or high-altitude cerebral edema. In this study, oxygen saturation decreased significantly, whereas heart rate and blood pressure increased after entering the plateau. These altitude-induced changes of physiological indices have a common trend, and they show no significant differences between AMS-S (ten subjects with the highest AMS scores) and AMS-R (ten subjects with the lowest AMS scores) groups. However, the subjects in the two different groups showed significantly different clinical features (Table 2).

### Metabolic Profiling and Differential Metabolites of Samples from High-altitude Post-exposure and Pre-exposure Groups

To ensure reproducibility of the analysis, the quality control (QC) samples were processed as real samples and then were randomly inserted among the real samples to be analysed 16 times. Multivariate analysis results of the QC samples showed the peak area deviation was <2SD, which demonstrated the robustness of the method (see Figure S2). This means that differences observed between groups by multivariate statistical analysis were more likely to reflect varied metabolite profiles rather than analytical variation.

**Figure 2:**
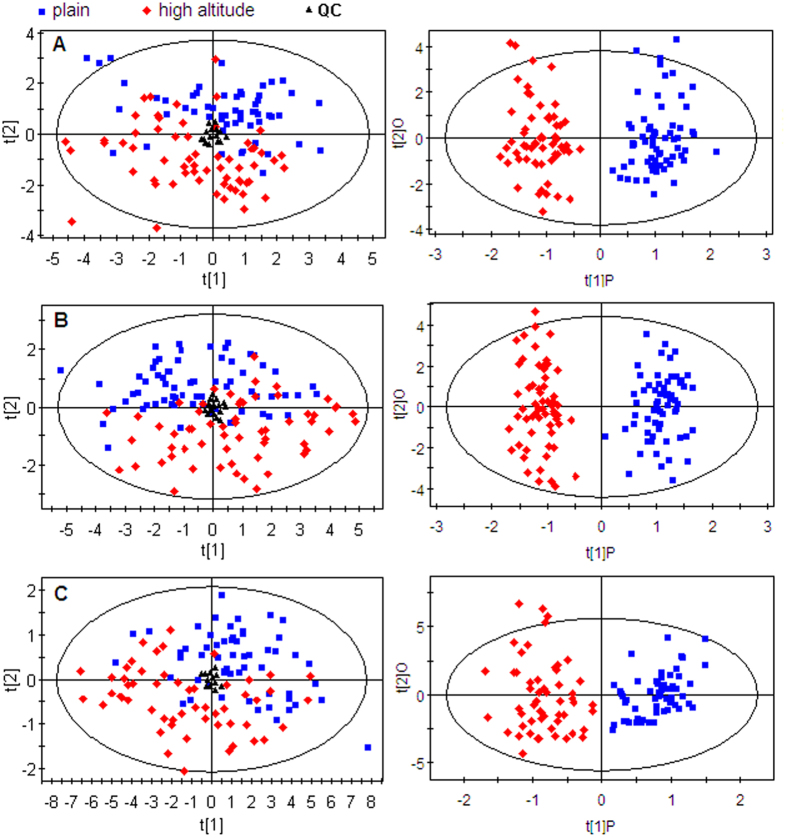
PCA and OPLS-DA scores plots of 60 subjects at high altitude (red diamonds) and on plain (blue boxes) based on plasma spectral data of (**A**) UPLC-QTOFMS positive ion mode, (**B**) UPLC-QTOFMS negative ion mode and (**C**) GC-MS.

Typical total ion current chromatograms in positive ion mode (ESI+) and negative ion mode (ESI−) of UPLC-QTOFMS and chromatograms of GC-MS obtained from a subject are shown in Figures S2 and S3, respectively, where marked variations can be visually observed between the two plasma chromatograms. A total of 799 peaks were obtained from UPLC-QTOFMS ESI+ mode (expressed as ESI+ data set) and 921 peaks obtained from ESI− mode (expressed as ESI− data set), whereas 129 peaks were obtained from GC-MS spectra (expressed as GC-MS data set). After data normalization, principal component analysis (PCA) was performed on the data set, which showed a trend of inter-group separation on the scores plots. Figure 2A–C illustrates PCA and orthogonal partial least squares-discriminant analysis (OPLS-DA) scores plots of 60 subjects at high altitude (red diamonds) and on plain (blue boxes) based on spectral data of UPLC-QTOFMS ESI+ mode (Fig. 2A); UPLC-QTOFMS ESI− mode (Fig. 2B); and GC-MS (Fig. 2C). All three OPLS-DA scores plots showed that the high-altitude post-exposure group were clearly separated from pre-exposure controls. The OPLS-DA models were validated by a permutation test (999 times). R intercept values of all models and their Q intercept values which are correlated with the extent of over-fitting were rather small, indicating that these models were satisfactory. Also, the *p*-values obtained from 7-fold cross-validation showed that all groups fitted by different models were significantly different (Table S1).

Fifty most significantly altered plasma metabolites (Table 3) in high-altitude post-exposure subjects relative to pre-exposure controls were identified from the OPLS-DA model of the GC-MS, ESI+ and ESI− spectral datasets, annotated by the mass of molecular and fragment ions, among which 17 were further validated by reference standards available in our laboratory.

### Metabolic Profiling and Differential Metabolites of Samples from the AMS-S and AMS-R Groups

Figure S4A–C illustrates PCA and OPLS-DA scores plots of the AMS-S group (red diamonds) and the AMS-R group (blue boxes) based on spectral data of UPLC-QTOFMS ESI+ mode (Figure S4A); UPLC-QTOFMS ESI− mode (Figure S4B); and GC-MS (Figure S4C). All three OPLS-DA scores plots showed that the AMS-S group was clearly separated from the AMS-R group.

**Figure 3:**
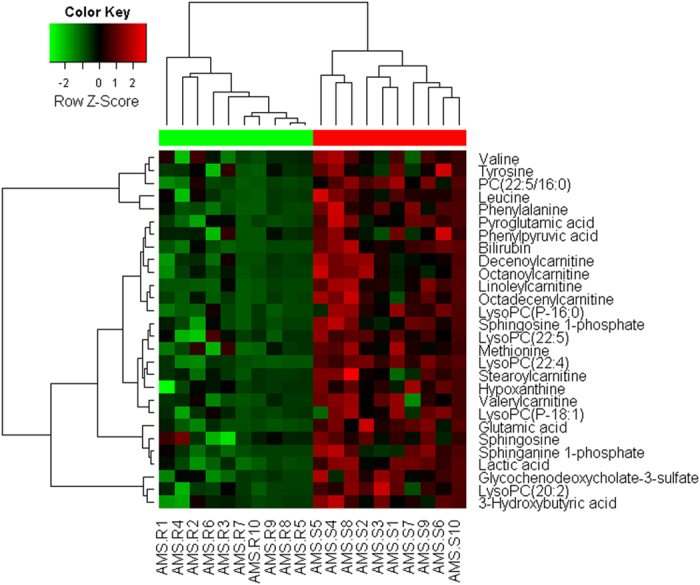
Heat map visualization based on the differential metabolites of importance for the plasma of the AMS-S group. Variable differences marked on the right corresponding to Table 2 are revealed between the AMS-S and AMS-R groups. Rows, differential metabolites; columns, samples; colour key indicates metabolite expression value, green is lowest and red is highest.

Twenty-eight most significantly altered plasma metabolites (Table 4) in the AMS-S group relative to the AMS-R group were identified from a two-component OPLS-DA model of the GC-MS, ESI+ and ESI− spectral data sets, annotated by the mass of molecular and fragment ions. A heat map, commonly used for unsupervised clustering, was constructed based on the potential candidates. The parallels heat map visualization (Fig. 3) using Ward’s method in computational systems analysis for the AMS-S and AMS-R groups showed distinct segregation.

### Metabolic Profiling and Differential Metabolites of Pre-exposure Samples from the AMS-S and AMS-R Groups

Figure S5A–C illustrates PCA and OPLS-DA scores plots of pre-exposure samples of AMS-S (Pre-AMS-S, red diamonds) and AMS-R (Pre-AMS-R, blue boxes) based on spectral data of UPLC-QTOFMS ESI+ mode (Figure S5A); UPLC-QTOFMS ESI− mode (Figure S5B); and GC-MS (Figure S5C). All three OPLS-DA scores plots showed Pre-AMS-S were clearly separated from Pre-AMS-R.

Five of the most significantly altered plasma metabolites (Table 5) in Pre-AMS-S relative to Pre-AMS-R were identified from the OPLS-DA model of the ESI+ and ESI− spectral datasets, annotated by the mass of molecular and fragment ions.

### Metabolic Pathway Analysis

**Figure 4:**
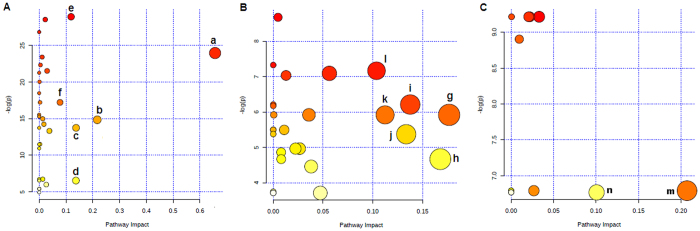
Summary of pathway analysis with MetaboAnalyst 3.0. (**A**) Altered metabolic pathways between high-altitude post-exposure and pre-exposure groups. a, linoleic acid metabolism; b, arachidonic acid metabolism; c, pyruvate metabolism; d, inositol phosphate metabolism; e, phenylalanine metabolism; f, citrate cycle. (**B**) Altered metabolic pathways between AMS-S and AMS-R groups. g, alanine, aspartate and glutamate metabolism; h, phenylalanine metabolism; i, pyruvate metabolism; j, sphingolipid metabolism; k, D-Glutamine and D-glutamate metabolism; l, glycerophospholipid metabolism. (**C**) Altered metabolic pathways between Pre-AMS-S and Pre-AMS-R groups. m, alanine, aspartate and glutamate metabolism; n, glycerophospholipid metabolism.

**Figure 5:**
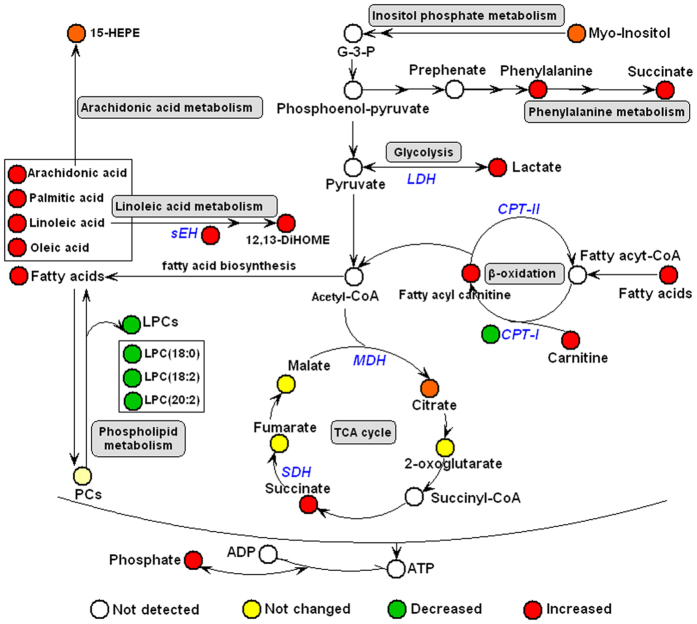
Schematic overview of the metabolites and major metabolic pathways as well as pathway-related enzyme changes in plasma of the 60 subjects after arriving at high altitude for four days. The metabolites and enzymes (italic) are shown in color: red represents increased metabolites or enzymes, green represents decreased metabolites or enzymes, yellow represents no change, and the open circles represent no detected metabolites.

Metabolite profiling focuses on the analysis of a group of metabolites related to a specific metabolic pathway in biological states. More detailed analysis of the relevant pathways and networks of AMS was performed by MetaboAnalyst which is a free, web-based tool that combines results from a powerful pathway enrichment analysis concerning the conditions under study. MetaboAnalyst, directed graph, uses the high-quality KEGG (Kyoto Encyclopedia of Genes and Genomes, www.genome.jp/kegg/) pathway database as its backend knowledgebase. Consequently, potential target metabolic pathway analysis (impact-value ≥0.10) with MetaboAnalyst revealed that metabolites which were identified together are important for the host response to high altitude, and are responsible for the metabolism of linoleic acid, arachidonic acid, pyruvate, inositol phosphate and phenylalanine (Fig. 4 and Table S2). Alanine, aspartate and glutamate metabolism, and the metabolism of each of phenylalanine, pyruvate, sphingolipid, glycerophospholipid, and D-glutamine and D-glutamate were found to be disturbed in the AMS-S group (Fig. 4 and Table S2). The top two metabolic pathways of importance, which were alanine, aspartate and glutamate metabolism and glycerophospholipid metabolism, were found to be disturbed in the Pre-AMS-S group (Fig. 4 and Table S2). Detailed related pathways were constructed using the reference map by searching KEGG (Fig. 5).

### The Levels of Key Enzymes Involved in the Pathways of Altered Metabolites

**Figure 6:**
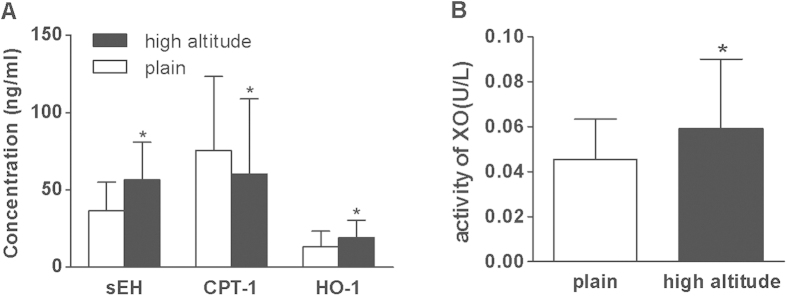
The levels of key enzymes involved in the pathways of altered metabolites. (**A**) The levels of key enzymes determined by ELISA. The error bars represent the S.D. of the mean. *p < 0.05, compared with plain control. Key: sEH, soluble epoxy hydrolase; CPT-I, carnitine palmitoyltransferase-I; HO-1, heme oxygenase-1. (**B**) The level of xanthine oxidase (XO) determined by a colorimetric method. The error bars represent the S.D. of the mean. *p < 0.05, compared with plain control.

To validate the metabolic changes, the levels of key enzymes in these metabolic pathways were determined by ELISA. As shown in Fig. 6, soluble epoxy hydrolase (sEH), xanthine oxidase (XO) and heme oxygenase-1 (HO-1), respectively involving in linoleic acid metabolism, phospholipid metabolism, purine metabolism and heme metabolism showed significant upregulation, whereas carnitine palmitoyltransferase-I (CPT-I) involved in fatty acid β-oxidation was downregulated in post-exposure samples compaired with pre-exposure samples.

## Discussion

The trend of contemporary scientific development is to follow systems biology. Identification of molecular markers and metabolic pathways has the potential to improve the understanding of pathophysiological mechanisms and the diagnosis and prognosis of a condition, as well as to identify the most efficacious therapy^11^,^12^,^13^. The process of acclimatization to high altitude is very complex; to date, the molecular mechanism involved and the pathogenesis of severe AMS remain unclear, and there are no reliable predictive biomarkers for AMS-S individuals. An approach using non-target metabolomics provides a global view of the organism and can be used to monitor the metabolic alterations that occur in different pathophysiological processes. In this study, we used a non-targeted mass spectrometry (MS)-based metabolomics approach to identify metabolic hemeatological biomarkers associated with the pathophysiological mechanism of early acclimatization to hypobaric hypoxia.

### The Interpretation of the Perturbed Metabolic Pathways after Exposure to High Altitude

To identify the relevant metabolic pathways involved in metabolic reprogramming during early acclimatization to high altitude, the differential metabolites were further subjected to MetaboAnalyst 3.0 and relevant pathways were identified. Based on our findings, an integrative view plot of the metabolic changes induced by hypobaric hypoxia was prepared (see Fig. 5). The major perturbed metabolic patterns and plausible pathways associated with hypobaric hypoxia are discussed below.

Of these perturbed metabolic pathways, linoleic acid metabolism is of particular note (Table S2). As shown in Table 3, linoleic acid and 12,13-DiHOME were significantly elevated at high altitude. It is well known that 12,13-DiHOME^14^ is produced in humans from linoleic acid, via its oxidation (by P450 monoxygenase) to the protoxin 12,13-EpOME (isoleukotoxin), followed by conversion to 12,13-DiHOME via the 12,13-EpOME by sEH. ELISA assay in this study showed that sEH was upregulated after exposure to high altitude, which supported the enhanced linoleic acid metabolism. Both 12,13-DiHOME and 12,13-EpOME are PPAR-γ ligands with potentially wide-ranging effects. In addition to its role as a PPAR ligand, 12,13-DiHOME exerts toxic and oxidative effects, inhibits mitochondrial function, stimulates neutrophil chemotactic activity and suppresses neutrophil respiratory burst activity^15^,^16^,^17^,^18^. Its role in AMS needs to be further studied.

Our results show that a number of lysophosphatidylcholines (LysoPCs) are obviously decreased and free fatty acids (FFA) (linoleic acid, arachidonic acid, eicosapentaenoic acid, docosahexaenoic acid, oleic acid, and palmitic acid) are increased after high-altitude exposure patients, and the reason is not clear. LysoPCs can mediate many cell-signalling pathways in monocytes/macrophages^19^,^20^ and specific receptors^21^, and therefore participate in the inflammatory response. The other product of PC metabolism, arachidonic acid, can be metabolized by cyclooxygenases and lipooxygenases, forming various eicosanoids such as prostaglandins, thromboxanes, leukotrienes and lipoxins that participate in the inflammatory response^22^. In this study, 15-HEPE, one product of arachidonic acid, is increased after high-altitude exposure, which may contribute to the severe AMS. In addition, a significant elevation of LysoPC in the AMS-S group might be responsible for AMS-S subjects being more vulnerable to hypoxia than AMS-R subjects during acclimatization to high altitude.

Uric acid is the end product of purine metabolism in humans and is generated by the action of the enzyme XO which catalyses the last two steps of uric acid conversion: hypoxanthine to xanthine and from xanthine to uric acid. Our observation of significantly increased levels of hypoxanthine and uric acid in the high-altitude group is consistent with previous reports^23^. The mechanism may relate to the reduction in adenosine triphosphate (ATP) levels with increased adenine nucleotide turnover coupled with activation of XO^24^. This hypothesis was verified by ELISA assay, which revealed significant upregulated activity of XO after high altitude exposure. Thus, inhibition of XO by allopurinol or other XO inhibitors would be expected to reduce uric acid production. Additionally, hypoxanthine was significantly increased in the AMS-S group compaired with that in the AMS-R group (Table 4), which suggests inhibition of XO would be expected to attenuate AMS.

Rising lactate suggests increased anaerobic glycolysis, whereas rising succinate and citrate suggest inhibited mitochondrial TCA cycle activity. It is well known that mitochondria can compensate for decreased energy production by increasing lactate production under hypoxia. Succinate is accumulated when the mitochondrial respiratory chain complex II switches from the use of succinate dehydrogenase to fumarate reductase during hypoxia^25^.

The carnitine system (including free carnitine and acylcarnitines) is essential for cell energy metabolism as a carrier of long-chain fatty acids for β-oxidation or as a reservoir pool of acyl-CoA^26^. In our study, the difference in the abundance of carnitines and their precursor, methionine, between pre- and post-exposure samples might reflect the alternative energy requirement associated with their acclimatization to high altitude. Other workers have reported that acute hypoxia caused the inhibition of FFA oxidation, which resulted in the accumulation of long-chain acylcarnitines^27^. Our results are in agreement with this report, and CPT-I as the key enzyme of FFA oxidation was significantly decreased and 10 long-chain acylcarnitines were significantly increased in the post-exposure group. In a different study, accumulation of these intermediates was shown to cause deleterious effects in cardiac tissue and decreased cardiac function^28^, which may contribute to maladaptation to high altitude. In our own study, in addition to long-chain acylcarnitines, free carnitine and two short-chain carnitines, acetyl-l-carnitine and propionyl-l-carnitine, were also increased in the post-exposure group, and the reason is not clear. Others have reported that l-carnitine supplementation attenuates intermittent hypobaric hypoxia-induced oxidative stress and delays muscle fatigue in rats^29^. Several previous studies indicated that administration of acetyl-l-carnitine attenuates neuronal damage, prevents apoptosis and improves energy status in hypoxic stress^30^,^31^. l-carnitine and propionyl-l-carnitine have been used in clinical treatment of cardiovascular diseases^32^. We speculate hypoxia can cause compensatory increase of l-carnitine and short-chain carnitines, which attenuate the damage of hypoxia by promoting β-oxidation of long-chain fatty acids to reduce the accumulation of toxic long-chain acylcarnitines. Our results also showed six long-chain acylcarnitines were significantly increased in the AMS-S group (Table 4), which may contribute to maladaptation to high altitude. Therefore, reducing the accumulation of toxic long-chain acylcarnitines may be important to attenuate AMS.

The low level of valine, a branched-chain amino acid (BCAA), was observed in the high-altitude post-exposure group. BCAAs may be an important alternative energy substrate. It seems that the reduction in ATP production due to the inhibition of the TCA cycle induced by hypobaric hypoxia as discussed above could lead to the utilization of BCAAs as alternative sources of energy. LC-MS and GC-MS spectra also showed changes in the metabolism of other α-amino acids, as represented by increased levels of alanine and proline in the high-altitude post-exposure group; α-amino acids are important energy metabolism precursors and can be transformed into some biomolecules, such as pyruvate, 2-oxoglutarate and fumarate, to enter the citrate cycle. One possible speculation is that high-altitude exposure leads to metabolic remodelling of α-amino acids to meet the energy requirement. In addition, the alterations in energy metabolism induced by high-altitude exposure were associated with ATP depletion and accumulation of phosphate. We speculate that supplements of the above-mentioned amino acids (valine, alanine and proline) may be helpful for improving energy metabolism and promoting acclimatization to high altitude.

Bile acids serve many important physiological functions, including cholesterol homeostasis, lipid absorption and generation of bile flow, that help in the excretion and recirculation of drugs, vitamins and endogenous and exogenous toxins^33^. In our own study, the bile acids tauroursodeoxycholic acid and glycochenodeoxycholate-3-sulfate were increased dramatically after high altitude exposure, and glycochenodeoxycholate-3-sulfate were significantly elevated in AMS-S group compaired with AMS-R group. Previous studies showed acute hypobaric hypoxia can cause significant damage of liver^34^ and gastrointestinal mucosa^35^. Therefore, the elevation of bile acids could be due in part to injury of liver and intestine and impaired bile acid enterohepatic circulation. A more recent study demonstrated bile acids can repress hypoxia-inducible factor 1α (HIF-1α) signalling and modulate the airway immune response^36^, which may have a significant influence on the progression and outcome of high-altitude respiratory disease. This result combined with data from previous studies indicate that rapid exposure to a hypoxic environment causes damage of gastrointestinal mucosa and the disruption of bile acid metabolism. Protecting gastrointestinal mucosal function may be of clinical importance in reducing the incidence of high-altitude diseases and improving treatment regimens.

As shown in Table 3, bilirubin was significantly elevated in the post-exposure group. It is conceivable that the elevation may be ascribed to the activation of HO-1 which typically catalyses the rate-limiting step in the heme salvage pathway, converting the prooxidant heme to biliverdin, which is then rapidly converted by biliverdin reductase to bilirubin. This hypothesis was verified by ELISA, which revealed significant upregulation of HO-1 after high altitude exposure. On the one hand, the increased bilirubin, serving as an antioxidant, may attenuate hypoxia-induced oxidative stress^37^,^38^. On the other hand, excessive bilirubin can enter the brain via the blood–brain barrier to induce neurologic damage^39^. In our experiments, bilirubin was significantly increased in the AMS-S group compared with the AMS-R group, which indicates the AMS-S group may have experienced stronger oxidative stress and neurologic damage.

Finally, changes in 3-hydroxybutyric acid and citrulline levels were also observed in post-exposure group, which was perplexing due to a lack of information about their biology pathways.

In conclusion, we have identified 44 significantly changed metabolites and predicted the major metabolites network by pattern recognition and pathway analysis. The identified target metabolites were found to encompass a variety of pathways related to inflammatory response-related metabolism (linoleic acid metabolism, arachidonic acid metabolism, phospholipid metabolism and purine metabolism), energy metabolism (glycolysis, TCA cycle, fatty acid metabolism and amino acid metabolism), bile acid metabolism and heme metabolism, which were helpful for revealing the complex mechanism of early acclimatization to high altitude.

### Potential Predictive Biomarkers for AMS-S Individuals

During early acclimatization to high altitude, some individuals are prone to show maladaptation and are more susceptible to AMS than others when exposed to identical hypoxia conditions, suggesting a possible genetic predisposition^40^. Many genes partly contribute to severe AMS pathogenesis, e.g. genes of the renin–angiotensin–aldosterone system, the heat-shock protein 70 family and endothelial nitric oxide synthase^41^,^42^,^43^. Our previous studies showed mitochondrial gene polymorphisms are also involved in the processes of acute impairment and chronic acclimatization of migrating lowlanders, as well as genetic adaptation of native residents at high altitudes^44^. Some individuals with certain mitochondrial DNA variations are susceptible to mountain sicknesses^45^. However, specific tests or biomarkers are sought after which would be more reliable and accessible for early prediction of AMS-S. In this study, we aimed to search for the potential predictive biomarkers for AMS-S using mebabolomics methods. We compared the pre-exposure plasma samples of AMS-S and AMS-R groups. Five most significantly altered plasma metabolites (Table 5) in Pre-AMS-S relative to Pre-AMS-R were identified. We infer that one or several combined metabolites of these metabolites could be predictive biomarkers for AMS-S individuals. Further research focusing on the validation of these potential markers is required, which may lead to new strategies for the prediction and treatment of AMS-S individuals.

## Conclusions

This is the first metabolomic study to determine plasma biochemical alterations of healthy individuals after four days at high altitude (5300 m) by using combined LC-MS and GC-MS. We have identified 50 significantly changed metabolites and predicted the major metabolites network by pattern recognition and pathway analysis. Combining the results from these methods, we have calculated several high confidence networks. The identified target metabolites were found to encompass a variety of pathways related to inflammatory response-related metabolism (linoleic acid metabolism, arachidonic acid metabolism, phospholipid metabolism and purine metabolism), energy metabolism (glycolysis, TCA cycle, fatty acid metabolism and amino acid metabolism), bile acid metabolism and heme metabolism. Identified differential metabolites and related pathways provide new insights for further understanding the pathophysiological mechanism of early acclimatization to hypobaric hypoxia and treatment of AMS-S individuals. Blocking or modifying these points of convergence are attractive approaches to promoting acclimatization to hypobaric hypoxia and the treatment of AMS-S individuals. Further studies are needed to deepen the understanding of the biological function and regulatory mechanism of the key metabolites and metabolic pathways. In addition, identified differential metabolites of plasma samples collected on plain may help the prediction of AMS-S individuals, which will be validated in future studies.

## Methods

### Subjects

Sixty healthy male volunteers participated in the study. Prior to the present experiment they had not been exposed to altitude. Their physical and physiological characteristics (mean ± SD) were: age 21.8 ± 1.8 years, height 172 ± 1 cm, body mass 65.8 ± 1.8 kg. Written informed consent was obtained from all subjects. The experimental protocol was reviewed and approved by the Ethical Committee of Third Military Medical University and was conducted according to the principles expressed in the Declaration of Helsinki.

### Experimental Procedures

The 60 subjects travelled by bus from the starting point of the study, at 1400 m, to an altitude of 5300 m over a 72-hour period, with a day’s rest at 3000 m. Daily measurements were made of blood pressure, heart rate and arterial oxygen saturation. All subjects consumed the same diet based on cooked meat, potatoes and boiled vegetables. Subjects did not receive medication for the duration of the study.

AMS symptoms were recorded from day 1 to day 5 using the Lake Louise Scoring (LLS) system^46^. Individuals with headaches and having total LLS >4 were considered to be AMS-susceptible (AMS-S) subjects, while subjects with LLS ≤4 or without headaches were considered to be AMS-resistant (AMS-R) subjects^46^. To maximize differences between AMS-S and AMS-R subjects, and avoid the inclusion of individuals with ambiguous AMS status, the subjects with the highest (top ten) and lowest (bottom ten) AMS scores, respectively named AMS-S and AMS-R groups, were included to explore the pathogenesis of severe AMS.

Fasting venous blood (with EDTA as an anticoagulant) was obtained from all subjects both on plain (1400 m) and on the fourth day after arriving at high altitude (5300 m). The plasma was separated immediately by centrifugation (3000 × *g*, 10 min). The harvested plasma samples were immediately frozen in dry ice and transported to Chongqing on dry ice by courier for further experiments.

### Chemicals and Reagents

Formic acid was obtained from Fluka (Buchs, Switzerland). Methoxylamine hydrochloride, N-methyl-N-(trimethylsilyl)-trifluoracetamide (MSTFA), pyridine, trimethyl-chlorosilane (TMCS), ammonium formate and citrate were purchased from Sigma-Aldrich (St. Louis, MO, USA). Methanol and acetonitrile (ACN) were chromatography pure (Merck, Germany). Lysophosphatidylcholine (18:0) were purchased from Larodan AB (Malmö, Sweden). Sphingosine was purchased from Acros Organics (NewJersey, USA). Lactate, sodium succinate, alanine, proline, phenylalanine, valine, leucine, methionine, tyrosine, glutamic acid, palmitic acid, oleic acid, linoleic acid, arachidonic acid, uric acid and hypoxanthine were obtained from Shanghai Jingchun Reagent Co. Ltd. Ultrapure water was prepared with a Milli-Q water purification system (Millipore, Bedford, MA, USA).

### Plasma Sample Preparation and Analysis by UPLC-QTOFMS

Plasma sample preparation and analysis with UPLC-QTOFMS were performed according to our published report^47^. A 100 μl aliquot of plasma sample was spiked with 10 μl L-2-chlorophenylalanine (1 mg/ml in water), followed by the addition of 400 μl of methanol/acetonitrile/acetone (1:1:1, v/v/v)^48^ into the tube. After vigorous shaking for 1 min and incubation on ice for 10 min, the mixture was centrifuged at 14000 × g for 15 min at 4 °C to precipitate the protein. The supernatant was filtered through a syringe filter (0.22 μm) and transferred into the sampling vial pending UPLC-QTOFMS analysis. Plasma samples from each group were alternated in random order in each analysis batch in order to avoid technical errors originating from sample preparation and sample analysis. As part of the system conditioning and quality control (QC) process, a pooled QC^49^ sample was prepared by mixing equal volumes (10 μl) of 120 samples.

UPLC-MS analysis was performed on an Agilent 1290 Infinity LC system coupled to Agilent 6530 Accurate-Mass Quadrupole Time-of-Flight (Q-TOF) mass spectrometer (Agilent, USA). Chromatographic separations were performed on an ACQUITY UHPLC HSS T3 C18 column (2.1 mm × 100 mm, 1.8 μm, Waters, Milford, Ireland) maintained at 45 °C. The flow rate was 400 μl/min and the injection volume was 4 μl. The mobile phase consisted of 0.1% formic acid (A) and ACN modified with 0.1% formic acid (B). A linear gradient was used as follows: 2% B at 0–3 min, 2%–95% B at 3–20 min, 95% B at 20–22 min and followed by re-equilibrated step of 5 min.

An electrospray ionization source interface was used, and was set in both positive and negative modes so as to monitor as many ions as possible. The optimized conditions were as follows: capillary voltage, 3.5 kV; drying gas flow, 11 l/min; gas temperature, 350 °C; nebulizer pressure, 45 psig; fragmentor voltage, 120 V; skimmer voltage, 60 V. Data were collected in centroid mode from 100 to 1100 m/z. Potential biomarkers were analysed by MS/MS in the Q-TOF. Nitrogen was used as the collision gas. MS/MS analysis was performed on the mass spectrometer set at different collision energy of 10–40 eV according to the stability of each metabolites. MS spectra were collected at 2 spectra/s, and MS/MS spectra were collected at 0.5 spectra/s, with a medium isolation window (~4 m/z). The same MS parameters was set in negative mode as that in positive mode.

### Plasma Sample Preparation and Analysis by GC-MS

Plasma samples were derivatized and subsequently analysed by GC-MS following our previously published protocols with minor modifications^50^. A 100 μl aliquot of plasma sample was spiked with two internal standards (10 μl L-2-chlorophenylalanine in water) and vortexed for 10 s, followed by the addition of 400 μl of methanol/acetonitrile/acetone (1:1:1, v/v/v)^48^ into the tube. After vigorous shaking for 1 min and incubation on ice for 10 min, the mixture was centrifuged at 14000 × g for 15 min at 4 °C to precipitate the protein. The supernatant (300 μl) was transferred into a GC vial and dried with a gentle nitrogen stream. The derivatization was performed using methoxyamine pyridine (75 μl; 15 mg/ml) at 37 °C for 1 h, followed by MSTFA (75 μl) with 1% TMCS as catalyst at 70 °C for 1 h.

The derivatized samples for GC-MS were analysed on a Thermo-Finnigan Trace DSQ fasting-scanning single-quadrupole MS (Thermo Electron Corporation) operated at unit mass resolving power. A 0.5 μl sample was injected with splitless mode to a TR-5MS column (30 m × 250 μm × 0.25 μm) with helium as the carrier gas at a flow of 1 ml/min. The injector temperature was set at 260 °C. The temperature of the ion source was adjusted to 200 °C and that of quadrupole was set at 150 °C. GC-MS was operated using electron impact ionization with a 60–600 atomic mass unit scan range. The initial temperature of the column was kept at 70 °C for 3 min. The temperature was ramped at 4 °C/min to 220 °C and then to 310 °C at a rate of 12 °C/min where it was held for 10 min.

### Metabolomic Data Preprocessing

Data preprocessing used the method previously published by our group with minor modifications^51^. The GC-MS and LC-MS data in an instrument-specific format were converted to NetCDF and mzData formats via Thermo Xconvert software and Agilent MassHunter Qualitative software, respectively. The program XCMS (version, 1.40.0) (http://metlin.scripps.edu/download/) was used for nonlinear alignment of the data in the time domain and automatic integration and extraction of the peak intensities^52^. XCMS parameters were default settings except for the following: full width at half maximum (fwhm) = 4, bandwidth (bw) = 5 and snthersh = 5 for GC-MS and fwhm = 10, bw = 10 and snthresh = 5 for UPLC-QTOFMS. The variables that did not present in at least of 80% groups were filtered^53^. The internal standard was used for data quality control (reproducibility) and data normalization. The ion peaks generated by the internal standard were also removed. The resulting three-dimensional matrix, including retention time and m/z pairs, sample names and normalized ion intensities, were introduced to multivariate data analysis.

### Multivariate Data Analysis

The three data sets resulting from GC-MS, UPLC-QTOFMS ESI+ and ESI− (expressed as G, P and N, respectively) were analysed and validated by multivariate statistical method, separately. Each data set was imported into the SIMCA-P 12.0 software package (Umetrics, Umeå, Sweden). Principal component analysis (PCA) and orthogonal partial least squares-discriminant analysis (OPLS-DA) were carried out to visualize the metabolic alterations between pre- and post-exposure in the 60 subjects after mean-centring and pareto-scaling, a technique that increases the importance of low-abundance ions without significant amplification of noise. In this study, the default 7-round cross-validation was applied with 1/7th of the samples being excluded from the mathematical model in each round, to guard against over-fitting. The variable importance in the projection (VIP) values of all the peaks from the 7-fold cross-validated OPLS-DA model were taken as a coefficient for peak selection. VIP ranks the overall contribution of each variable to the OPLS-DA model, and those variables with VIP >1.0 are considered relevant for group discrimination^54^. Herein, VIP statistics and S-plot were applied to obtain the significant variables for subsequent metabolic pathway analysis^55^.

### Biomarkers Screening and Chemical Elucidation

Significantly characteristic differential metabolites or metabolic features between pre- and post-exposure groups were screened using the S-plot of the OPLS-DA model. The Paired t-test was selected to measure the significance of each metabolite in separating post-exposure group from pre-exposure group. The Student’s t-test was selected to measure the significance of each metabolite in separating the AMS-S group from the AMS-R group at high altitude, and the corresponding pre-exposure samples. The differences were considered significant when p <0.05. To account for multiple comparisons, the false discovery rate was estimated as the maximum *q* value^56^ in the set of significant differences for the metabolomic data set. False discovery rates were computed using the R package (http://www.r-project.org/). The significance of the group differences was evaluated by the *p* value for the fixed-effect parameter estimate of group differences.

Metabolite identification from these selected peaks was performed separately. GC-MS metabolites were identified by comparing the mass fragments with the NIST database installed in the Thermo-Finnigan Trace DSQ GC/MS system with a similarity of more than 70% and finally verified by available reference compounds. Metabolites obtained from positive and negative ion modes of the UPLC-QTOFMS analysis were identified as our previous work^57^. Briefly, the quasi-molecular ion peak was found according to the accurate mass and retention time in the extracted ion chromatogram (EIC), and then the most probable molecular formula were calculated by Agilent MassHunter software. For example, MS/MS analysis of m/z 162.1123 was performed using UPLC-QTOFMS in the same chromatographic and mass spectrometric conditions as that in MS spectrum. With its fragmentation and the databases such as METLIN (http://metlin.scripps.edu) and HMDB (http://www.hmdb.ca/), the major fragment ions m/z 103.0392, 85.0287 and 60.0810 represent the fragments of [C_7_H_15_NO_3_]^+^, [C_4_H_5_O_2_]^+^ and [C_3_H_9_N]^+^, respectively. Therefore, the m/z 162.1123 was identified as carnitine according to the elemental composition, retention time and fragmentation information. Finally, the MS/MS spectrum of the commercial standard was used to confirm the identified compound. Other biomarkers were similarly identified and listed in Tables 3, 4, 5, and the structures and MS/MS spectra of the metabolites were presented in Supporting Information Figures S6–S7.

Pathway analysis and visualization using the KEGG (www.genome.jp/kegg/) pathway database was carried out using Metaboanalyst^58^,^59^.

### The Determination of Key Enzymes in Relevant Metabolic Pathways

The levels of sEH, CPT-I and HO-1 were determined by ELISA assays in all plasma samples using commercially available kits (Nanjing Jiancheng Bioengineering Institute, China) as manufacturer’s instructions. The activity of XO were determined by a colorimetric method using commercially available kits (Nanjing Jiancheng Bioengineering Institute, China).

## Additional Information

**How to cite this article**: Liao, W.-T. *et al*. Metabolite Modulation in Human Plasma in the Early Phase of Acclimatization to Hypobaric Hypoxia. *Sci. Rep.* **6**, 22589; doi: 10.1038/srep22589 (2016).

## Supplementary Material

Supplementary Information

## Figures and Tables

**Figure 1:**
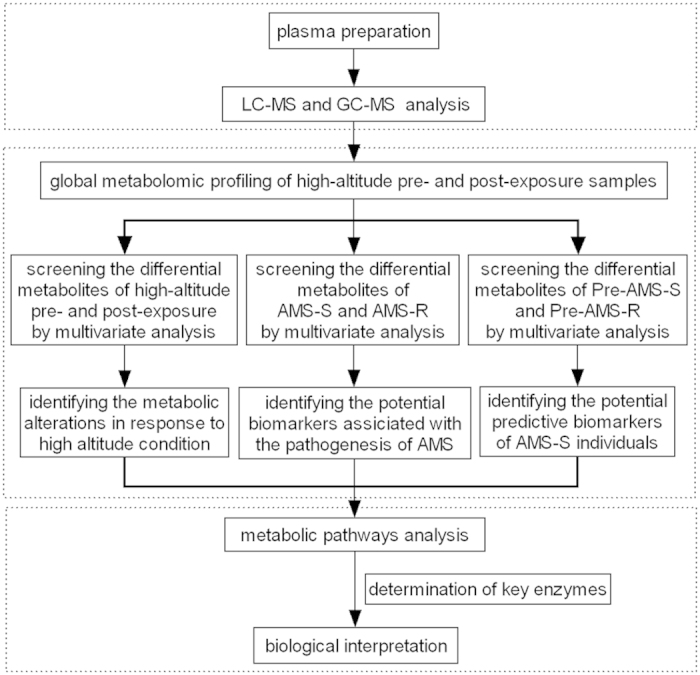
Schematic flow chart of the metabolic profiling strategy used in this study. AMS-S, acute mountain sickness susceptible subjects; AMS-R, acute mountain sickness resistant subjects; Pre-AMS-S, pre-exposure samples of AMS-S subjects; Pre-AMS-R, pre-exposure samples of AMS-R subjects.

**Table 1:** Basic physiological data^a^ based on 60 subjects.

Variables	Plain	High Altitude				
		Day 1	Day 2	Day 3	Day 4	Day 5
Altitude, m	1400	5300	5300	5300	5300	5300
Oxygen saturation, %	96.7 (1.3)	79.6 (6.6)	75.1 (9.1)	77.3 (7.3)	77.9 (6.0)	79.0 (5.0)
Heart rate, bpm	67 (10)	94 (11)	93 (16)	93 (16)	98 (14)	92 (13)
Lake Louise score	0 (0–0)	5 (3–6)	6 (3–8)	5 (2–6.5)	4 (2–6)	3 (1.5–4)
Headaches score	0 (0–0)	1 (1–2)	2 (1–2)	1 (1–2)	1 (0–1)	0 (0–1)
Blood pressure, mm Hg	114/62 (13/10)	119/73 (12/9)	122/72 (13/10)	121/71 (12/11)	123/71 (13/10)	121/70 (9/8)

^a^Data are presented as mean (SD) or median (IQR) unless otherwise indicated.

**Table 2:** Basic physiological data^a^ based on ten acute mountain sickness susceptible (AMS-S) subjects and ten acute mountain sickness resistant (AMS-R) subjects.

Variables	Group	Plain	High Altitude				
			Day 1	Day 2	Day 3	Day 4	Day 5
Altitude, m		1400	5300	5300	5300	5300	5300
Oxygen saturation, %	AMS-S	96.4(1.4)	79.0 (4.7)	74.8 (8.3)	78.5 (4.0)	76.4 (7.0)	79.7 (4.0)
	AMS-R	97.1 (0.7)	81.4 (4.2)	80.9 (3.6)	81.7 (2.6)	81.2 (3.0)	80.3 (6.0)
Heart rate, bpm	AMS-S	68 (9)	91 (13)	91 (22)	96 (20)	100 (16)	94 (15)
	AMS-R	66 (11)	91 (10)	88 (17)	87 (13)	92 (13)	88 (13)
Lake Louise score	AMS-S	0 (0–0)	7 (6–11)**	10 (8–12)**	6 (5–9)**	7 (5–7)**	3 (3–6)*
	AMS-R	0 (0–0)	2 (1–2.75)	2 (0–2.75)	1 (0–1)	0.5 (0–2)	1 (0–3)
Headaches score	AMS-S	0 (0–0)	1 (1–2)*	2 (2–3)**	2 (1–2)**	1 (1–2)**	1 (0–1)**
	AMS-R	0 (0–0)	1 (0–1)	1 (0–1)	0.5 (0–1)	0 (0–0)	0 (0–0)
Blood pressure, mm Hg	AMS-S	114/59 (12/9)	121/71 (14/13)	122/69 (14/12)	119/65 (10/10)	125/73 (13/9)	118/68 (10/7)
	AMS-R	114/61 (12/7)	125/77 (9/8)	120/75 (11/8)	124/75 (11/14)	124/74 (14/7)	122/72 (9/9)

^a^Data are presented as mean (SD) or median (IQR). *p < 0.05, **p < 0.01 compared with AMS-R.

**Table 3:** Summary of the differentially expressed plasma metabolites in 60 subjects at high altitude relative to plain.

Metabolites	Metabolic pathway	VIP^b^	%RSD^c^	p-value^d^	q-value	Ratio (high altitude/plain)
ESI+						
Carnitine^a^	Fatty acid transportation	2.52	9.0	4.66E-05	3.74E-04	1.25
Acetylcarnitine^a^	Fatty acid transportation	2.55	7.4	1.44E-07	2.56E-06	1.51
Propionylcarnitine	Fatty acid transportation	1.47	7.9	2.92E-05	2.36E-04	1.33
Octenoylcarnitine	Fatty acid transportation	1.33	6.8	1.21E-09	3.14E-08	1.53
Decanoylcarnitine	Fatty acid transportation	2.84	8.3	1.02E-06	1.24E-05	1.76
Dodecenoylcarnitine	Fatty acid transportation	1.91	9.7	1.38E-08	2.33E-07	1.76
Dodecanoylcarnitine	Fatty acid transportation	1.91	5.3	4.20E-07	4.92E-06	1.79
Tetradecadienylcarnitine	Fatty acid transportation	2.16	7.7	1.80E-07	2.77E-06	2.05
Tetradecenoylcarnitine	Fatty acid transportation	2.34	10.8	2.08E-08	2.94E-07	2.34
Tetradecanoylcarnitine	Fatty acid transportation	1.12	6.2	1.02E-08	2.09E-07	1.78
Hexadecenoylcarnitine	Fatty acid transportation	1.35	8.4	2.51E-11	6.24E-10	2.02
Octadecenoylcarnitine	Fatty acid transportation	2.78	10.6	8.08E-11	1.40E-09	1.7
C20:1-carnitine	Fatty acid transportation	1.30	9.4	7.43E-12	1.33E-10	1.69
LysoPC(18:2)	Phospholipid metabolism	7.84	9.3	9.14E-04	4.49E-03	0.62
LysoPC(20:2)	Phospholipid metabolism	2.31	7.2	8.19E-15	1.04E-13	0.63
LysoPC(18:0)^a^	Phospholipid metabolism	2.56	7.4	1.55E-13	3.57E-11	0.65
Glycerophosphocholine	Phospholipid metabolism	1.43	9.1	3.00E-08	9.70E-07	0.57
Valine^a^	Valine metabolism	3.38	5.7	2.86E-06	4.43E-05	0.64
Proline^a^	Proline metabolism	1.00	7.0	6.39E-03	2.26E-02	1.23
Methionine^a^	Methionine metabolism	1.62	7.2	3.86E-07	4.63E-06	1.17
Uric acid^a^	Purine metabolism	1.52	3.0	1.51E-03	9.87E-03	1.2
Hypoxanthine^a^	Purine metabolism	1.27	6.7	3.10E-04	1.53E-03	1.58
Phenylalanine^a^	Phenylalanine metabolism	3.41	4.6	7.22E-05	6.25E-04	1.13
Bilirubin	Heme metabolism	3.19	4.2	4.76E-10	1.12E-08	3.01
ESI−						
Citrulline^a^	Urea cycle	1.19	6.8	1.76E-10	9.56E-09	0.66
Deoxyribose 1-phosphate	Pyrimidine Metabolism	1.69	7.9	3.54E-07	4.41E-06	0.57
Glycochenodeoxycholate-3-sulfate	Bile acid metabolism	1.01	3.8	2.76E-03	1.07E-02	1.28
Tauroursodeoxycholic acid	Bile acid metabolism	1.10	9.2	2.03E-03	8.41E-03	2.36
Tetradecenoic acid	Fatty acid metabolism	1.29	4.4	2.08E-06	2.08E-05	1.79
12,13-DiHOME	Linoleic acid metabolism	1.14	8.3	3.20E-07	4.04E-06	1.53
Eicosapentaenoic acid^a^	Fatty acid metabolism	1.27	9.0	1.06E-06	1.14E-05	1.94
Docosahexaenoic acid^a^	Fatty acid metabolism	1.45	8.1	3.44E-05	2.37E-04	1.27
15-HEPE	Arachidonic acid metabolism	1.85	6.2	1.02E-05	8.06E-05	2.48
GC-MS						
Lactic acid^a^	Glycolysis	6.67	11.6	4.31E-04	2.13E-03	1.24
Succinic acid	TCA cycle	1.46	9.8	1.01E-10	2.64E-09	1.33
Citric acid^a^	TCA cycle	1.57	7.6	8.13E-04	6.07E-03	1.33
Palmitic acid^a^	Fatty acid metabolism	2.87	8.3	3.29E-06	3.06E-05	1.91
Oleic acid^a^	Fatty acid metabolism	4.47	7.5	2.73E-09	7.40E-08	2.43
Linoleic acid^a^	Linoleic acid metabolism	4.33	10.7	8.03E-09	1.85E-07	1.84
Arachidonic acid^a^	Arachidonic acid metabolism	2.61	5.6	2.64E-08	4.68E-07	1.59
Alanine^a^	Amino acid metabolism	1.01	3.5	2.43E-05	1.07E-04	1.24
Myo-inositol	Inositol phosphate metabolism	1.01	7.1	5.25E-04	3.03E-03	1.27
3-Hydroxybutyric acid	Others	2.01	10.9	3.80E-05	2.84E-04	2.44
Phosphoric acid	Others	1.16	5.8	4.13E-06	4.15E-05	1.71

^a^Metabolites validated by reference standards. The others were putatively annotated metabolites.

^b^Variable Importance in Projection.

^c^Variation of the metabolites concentrations in QC samples expressed as relative standard deviation (%RSD).

^d^Paired t-test.

**Table 4:** Summary of the differentially expressed plasma metabolites in patients of AMS-S relative to AMS-R.

Metabolites	Metabolic pathway	VIP^b^	%RSD^c^	p-value^d^	q-value	Ratio (AMS-S/AMS-R)
ESI+						
Valerylcarnitine	Fatty acid transportation	1.63	5.1	1.97E-03	1.01E-02	1.51
Octanoylcarnitine	Fatty acid transportation	1.85	8.3	6.78E-03	3.87E-02	1.88
Decenoylcarnitine	Fatty acid transportation	2.20	5.1	3.54E-03	1.87E-02	1.94
Linoleylcarnitine	Fatty acid transportation	2.70	9.9	3.22E-04	4.03E-03	1.71
Octadecenylcarnitine	Fatty acid transportation	2.48	10.6	6.97E-04	7.23E-03	1.71
Stearoylcarnitine	Fatty acid transportation	1.51	5.9	5.65E-03	3.25E-02	1.54
LysoPC(22:5)	Phospholipid metabolism	1.93	8.2	5.14E-04	5.81E-03	1.54
LysoPC(P-16:0)	Phospholipid metabolism	2.27	7.9	2.76E-04	3.89E-03	1.45
LysoPC(22:4)	Phospholipid metabolism	1.94	7.3	1.78E-04	2.90E-03	2.06
LysoPC(P-18:1)	Phospholipid metabolism	1.42	4.6	2.88E-03	1.52E-02	1.53
PC(38:5)	Phospholipid metabolism	4.24	8,9	3.89E-04	4.21E-03	1.96
Sphingosine^a^	Sphingolipid metabolism	1.05	6.0	5.32E-03	3.15E-02	1.30
Sphingosine 1-phosphate	Sphingolipid metabolism	1.79	5.7	2.51E-03	1.44E-02	1.37
Sphinganine 1-phosphate	Sphingolipid metabolism	1.15	7.7	7.22E-04	7.50E-03	1.52
Glutamic acid^a^	Glutamate metabolism	1.07	8.0	6.13E-04	6.40E-03	1.54
Methionine^a^	Methionine metabolism	1.40	7.2	8.03E-04	8.55E-03	1.14
Hypoxanthine^a^	Purine metabolism	1.86	6.7	9.38E-04	8.84E-03	1.88
Pyroglutamic acid^a^	Glutathione metabolism	1.91	5.8	4.51E-04	4.72E-03	1.33
Phenylpyruvic acid	Phenylalanine metabolism	1.55	7.0	4.46E-03	2.69E-02	1.17
Phenylalanine^a^	Phenylalanine metabolism	4.01	4.6	4.12E-04	4.35E-03	1.28
Bilirubin	Heme metabolism	2.19	4.2	5.14E-03	3.11E-02	2.49
ESI−						
Glycochenodeoxycholate-3-sulfate	Bile acid metabolism	2.22	3.8	3.91E-04	3.20E-03	2.16
LysoPC(20:2)	Phospholipid metabolism	1.39	3.8	3.41E-03	2.54E-02	1.59
GC-MS						
Lactic acid^a^	Glycolysis	1.29	11.6	3.97E-05	2.42E-04	1.45
Valine^a^	Valine metabolism	2.82	7.4	3.67E-03	2.55E-02	1.25
Leucine^a^	Leucine metabolism	4.89	5.6	4.08E-04	2.98E-03	1.31
Tyrosine^a^	Tyrosine metabolism	2.32	8.5	3.04E-03	2.22E-02	1.17
3-Hydroxybutyric acid	Others	2.12	10.9	5.53E-03	3.88E-02	2.02

^a^Metabolites validated by reference standards. The others were putatively annotated metabolites.

^b^Variable Importance in Projection.

^c^Variation of the metabolites concentrations in QC samples expressed as relative standard deviation (%RSD).

^d^Student’s t-test.

**Table 5:** Summary of the differentially expressed plasma metabolites in patients of Pre-AMS-S relative to Pre-AMS-R.

Metabolites	Metabolic pathway	VIP^b^	%RSD^c^	p-value^d^	q-value	Ratio (Pre-AMS-S/Pre-AMS-R)
ESI+						
Sphingomyelin(d18:1/16:0)	Sphingolipid metabolism	4.40	7.4	4.27E-04	5.69E-03	2.59
Phosphatidylcholine(38:5)	Phospholipid metabolism	3.04	6.8	2.97E-03	2.70E-02	2.36
Glutamic acid^a^	Glutamine and glutamate metabolism	1.01	8.0	1.23E-03	1.26E-02	2.10
Glyceric acid^a^	Glycine and Serine Metabolism	1.94	4.4	1.35E-04	2.13E-03	1.82
ESI−						
12,13-DiHOME	Fatty acid metabolism	1.33	8.3	2.83E-03	2.54E-02	1.73

^a^Metabolites validated by reference standards. The others were putatively annotated metabolites.

^b^Variable Importance in Projection.

^c^Variation of the metabolites concentrations in QC samples expressed as relative standard deviation (%RSD).

^d^P means p value obtained from Student’s t-test.
