# Supplementary material for: Metabolite Modulation in Human Plasma in the Early Phase of Acclimatization to Hypobaric Hypoxia
Source: Sci Rep. 2016 Mar 4;6:22589. doi: 10.1038/srep22589 (PMC4778071; doi:10.1038/srep22589)
Supplement: Supplementary Information [file srep22589-s1.doc]

**Metabolite Modulation in Human Plasma in the Early Phase of Acclimatization to** **Hypobaric Hypoxia**

**Authors**

Wenting Liao^1,4,5^, Bao Liu^1,4,5^, Jian Chen^2,4,5^, Jianhua Cui^6^, Yixing Gao^1,4,5^, Fuyu Liu^1,4,5^, Gang Xu^1,4,5^, Bingda Sun^1,4,5^, Erlong Zhang^1,4,5^, Zhibin Yuan^1,4,5^, Gang Zhang^3,4,5*^, Yuqi Gao1,4,5[[1]](#footnote-2)

**Supplemental Tables**

| **Table S1.** Summary of parameters for assessment of the quality of PCA and OPLS-DA models | | | | | | | | | |
| --- | --- | --- | --- | --- | --- | --- | --- | --- | --- |
|  |  | models | no.*^a^* | R^2^X_cum_*^b^* | R^2^Y_cum_*^b^* | Q^2^Y_cum_*^b^* | R intercept *^c^* | Q intercept *^c^* | *p^d^* |
| High altitude *vs.* plain | | | | | | | | | |
|  | LC-MS (ES+) | PCA | 2 | 0.393 | - | 0.340 | - | - | - |
|  |  | PLS-DA | 3 | 0.407 | 0.907 | 0.819 | 0.390 | -0.212 | 0.000 |
|  |  | OPLS-DA | 1+2 | 0.407 | 0.907 | 0.825 | - | - | 0.000 |
|  | LC-MS (ES-) | PCA | 2 | 0.439 | - | 0.397 | - | - | - |
|  |  | PLS-DA | 3 | 0.475 | 0.912 | 0.736 | 0.425 | -0.115 | 0.000 |
|  |  | OPLS-DA | 1+2 | 0.475 | 0.912 | 0.744 | - | - | 0.000 |
|  | GC-MS | PCA | 2 | 0.476 |  | 0.441 | - | - |  |
|  |  | PLS-DA | 3 | 0.479 | 0.774 | 0.670 | 0.351 | -0.117 | 0.000 |
|  |  | OPLS-DA | 1+2 | 0.479 | 0.774 | 0.649 | - | - | 0.000 |
| AMS-S *vs.* AMS-R |  |  |  |  |  |  |  |  |  |
|  | LC-MS (ES+) | PCA | 2 | 0.471 | - | 0.189 | - | - | - |
|  |  | PLS-DA | 2 | 0.416 | 0.908 | 0.768 | 0.553 | -0.148 | 0.006 |
|  |  | OPLS-DA | 1+1 | 0.416 | 0.908 | 0.743 | - | - | 0.003 |
|  | LC-MS (ES-) | PCA | 2 | 0.489 | - | 0.225 | - | - | - |
|  |  | PLS-DA | 2 | 0.480 | 0.896 | 0.772 | 0.592 | -0.106 | 0.004 |
|  |  | OPLS-DA | 1+1 | 0.480 | 0.896 | 0.751 | - | - | 0.002 |
|  | GC-MS | PCA | 2 | 0.592 |  | 0.429 | - | - | - |
|  |  | PLS-DA | 3 | 0.635 | 0.943 | 0.749 | 0.629 | -0.105 | 0.032 |
|  |  | OPLS-DA | 1+2 | 0.635 | 0.943 | 0.619 | - | - | 0.020 |
| Pre-AMS-S *vs*. Pre-AMS-R | | | | | | | | | |
|  | LC-MS (ES+) | PCA | 2 | 0.452 | - | 0.198 | - | - | - |
|  |  | PLS-DA | 2 | 0.417 | 0.916 | 0.822 | 0.579 | -0.114 | 0.002 |
|  |  | OPLS-DA | 1+1 | 0.417 | 0.916 | 0.773 | - | - | 0.001 |
|  | LC-MS (ES-) | PCA | 2 | 0.498 | - | 0.264 | - | - | - |
|  |  | PLS-DA | 2 | 0.430 | 0.946 | 0.621 | 0.599 | -0.101 | 0.027 |
|  |  | OPLS-DA | 1+1 | 0.430 | 0.946 | 0.650 | - | - | 0.005 |
| *^a^* No. is the number of components. *^b^* R^2^X_cum_ and R^2^Y_cum_ are the cumulative modeled variation in X and Y matrix, respectively, and Q^2^Y_cum_ is the cumulative predicted variation in Y matrix. *^c^* R and Q were obtained after permutation test (n = 999). *^d^* *P is p* value obtained from cross validation ANOVA of PLS-DA.or OPLS-DA | | | | | | | | | |
|
|

| **Table S2.** Result from Metabolic Pathway Analysis with MetaboAnalyst 3.0^a^ | | | | | | |
| --- | --- | --- | --- | --- | --- | --- |
| no. | pathway name | total cmpd | hits | raw *p* | -log(p) | impact |
| High altitude *vs.* plain | | | | | | |
| 1 | Linoleic acid metabolism | 15 | 2 | 3.91E-11 | 23.965 | 0.6563 |
| 2 | Arachidonic acid metabolism | 62 | 2 | 3.57E-07 | 14.847 | 0.2167 |
| 3 | Pyruvate metabolism | 32 | 1 | 1.08E-06 | 13.743 | 0.1376 |
| 4 | Inositol phosphate metabolism | 39 | 1 | 1.45E-03 | 6.539 | 0.1370 |
| 5 | Phenylalanine metabolism | 45 | 2 | 2.76E-13 | 28.920 | 0.1191 |
| 6 | Citrate cycle (TCA cycle) | 20 | 2 | 3.32E-08 | 17.220 | 0.0777 |
| AMS-S *vs.* AMS-R | | | | | | |
| 1 | Alanine, aspartate and glutamate metabolism | 24 | 1 | 2.69E-03 | 5.920 | 0.1766 |
| 2 | Phenylalanine metabolism | 45 | 3 | 9.38E-03 | 4.669 | 0.1678 |
| 3 | Pyruvate metabolism | 32 | 1 | 2.00E-03 | 6.212 | 0.1376 |
| 4 | Sphingolipid metabolism | 25 | 3 | 4.64E-03 | 5.373 | 0.1335 |
| 5 | D-Glutamine and D-glutamate metabolism | 11 | 1 | 2.69E-03 | 5.920 | 0.1123 |
| 6 | Glycerophospholipid metabolism | 39 | 2 | 7.72E-04 | 7.166 | 0.1037 |
| Pre-AMS-S *vs.* Pre-AMS-R | | | | | | |
| 1 | Alanine, aspartate and glutamate metabolism | 24 | 1 | 1.12E-03 | 6.790 | 0.2070 |
| 2 | Glycerophospholipid metabolism | 39 | 1 | 1.15E-03 | 6.766 | 0.1005 |
| *^a^*Total is the total number of compounds in the pathway; the hits is the actually matched number from the user uploaded data; the raw *p* is the original *p* value calculated from the enrichment analysis; the impact is the pathway impact value calculated from pathway topology analysis. | | | | | | |
|
|

**Supplemental Figures**


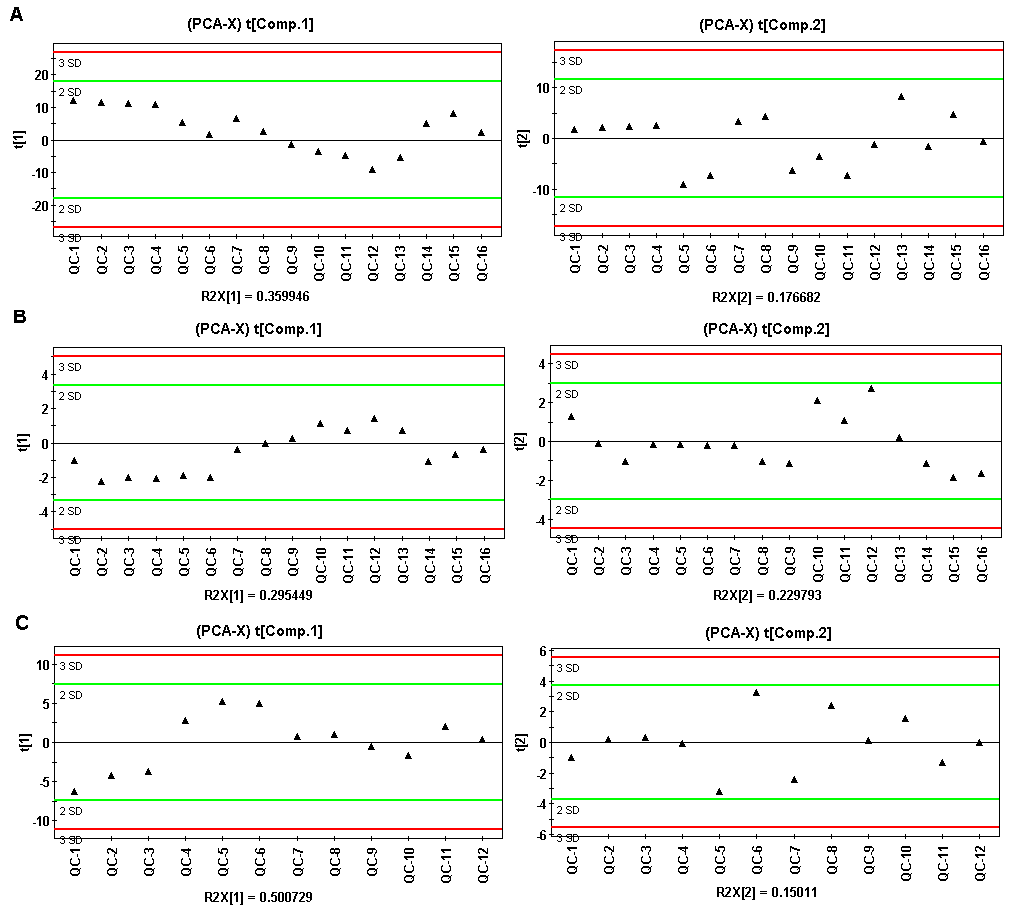


**Figure S1.** Line plots of QC samples generated by PCA using component 1 and 2. Peak area deviation could be evaluated by distribution of the runs. X-axis, run order; Y-axis, standard deviation. QC plots for the first and second component from (A) UPLC-QTOFMS positive ion mode data; (B) UPLC-QTOFMS negative ion mode data; (C) GC-MS.


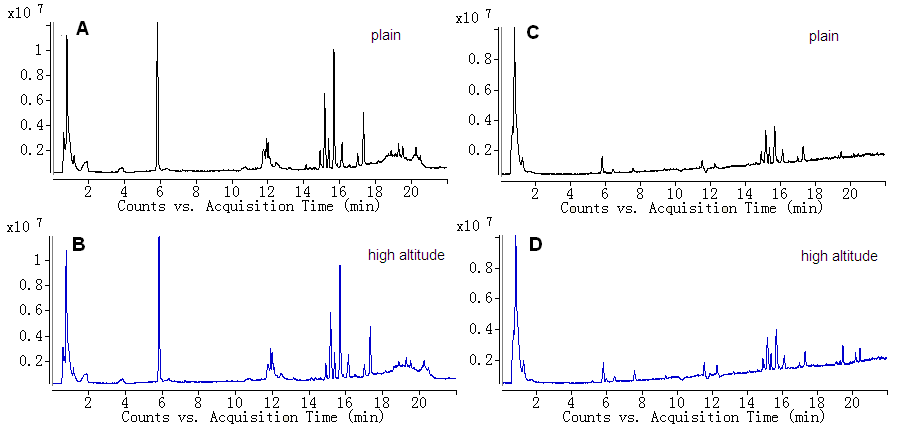


**Figure S2.** Typical total ions current chromatograms from plasma samples separated on

LC (ESI+, A and B) and LC (ESI-, C and D).

**
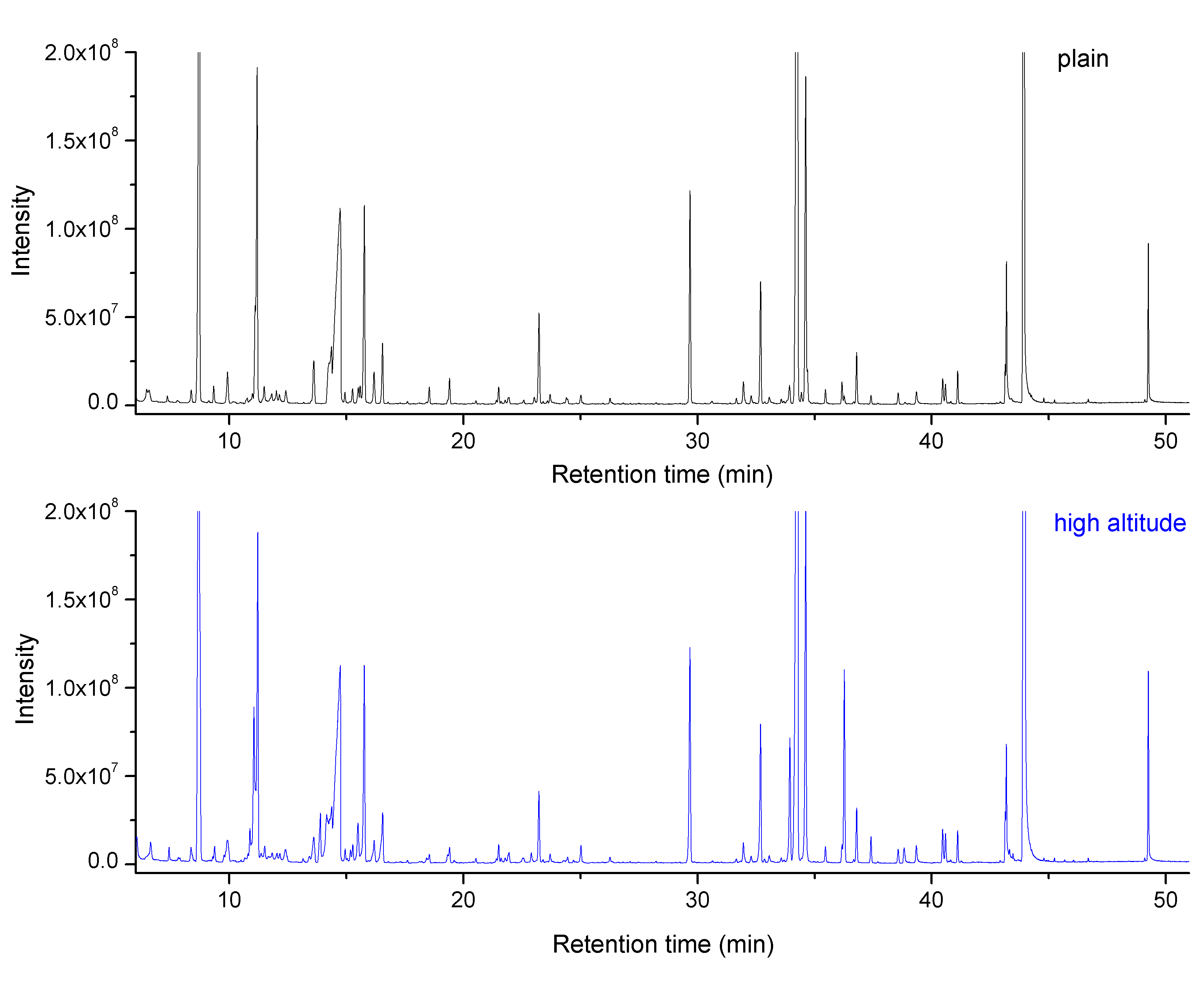
**

**Figure S3.** Typical total ions current chromatograms from plasma samples separated on GC.

**
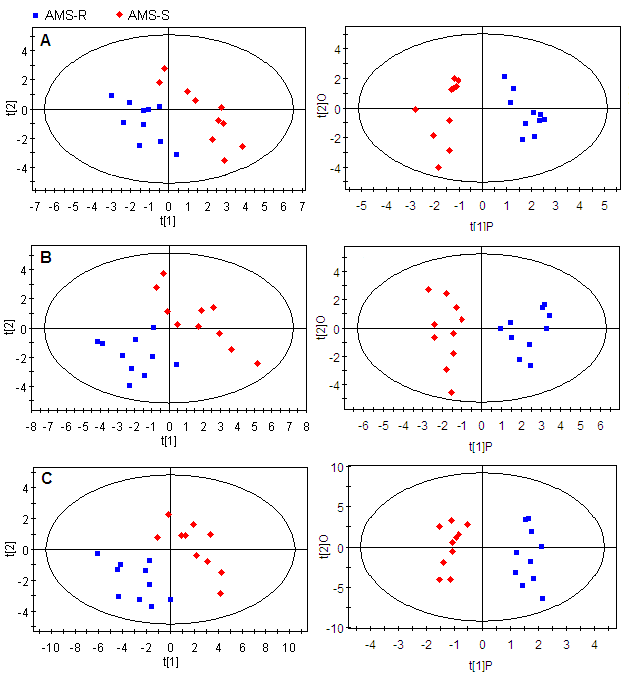
**

**Figure S4.** PCA and OPLS-DA scores plots of AMS-S (red diamonds) and AMS-R (blue boxes) groups at high altitude based on plasma spectral data of (A) UPLC-QTOFMS positive ion mode, (B) UPLC-QTOFMS negative ion mode and (C) GC-MS.

**
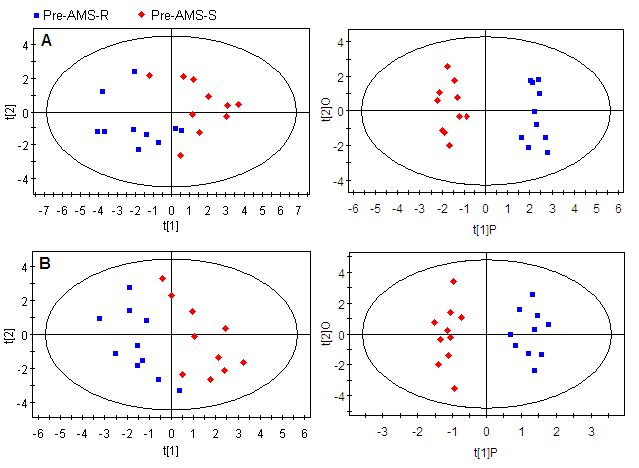
**

**Figure S5.** PCA and OPLS-DA scores plots s of pre-exposure samples from the AMS-S group (Pre-AMS-S, red diamonds) and AMS-S group (Pre-AMS-R, blue boxes) based on plasma spectral data of (A) UPLC-QTOFMS positive ion mode and (B) UPLC-QTOFMS negative ion mode.


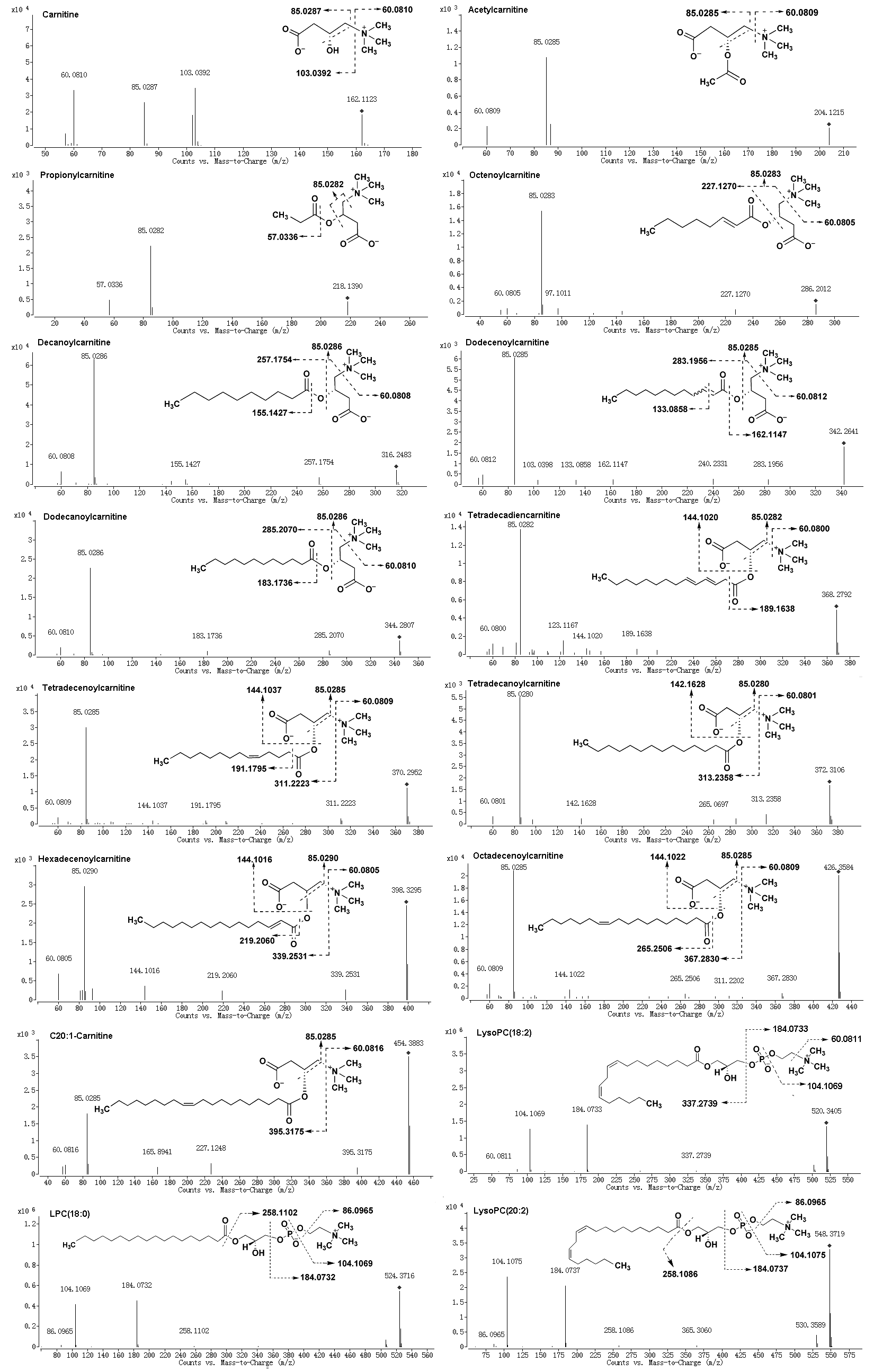


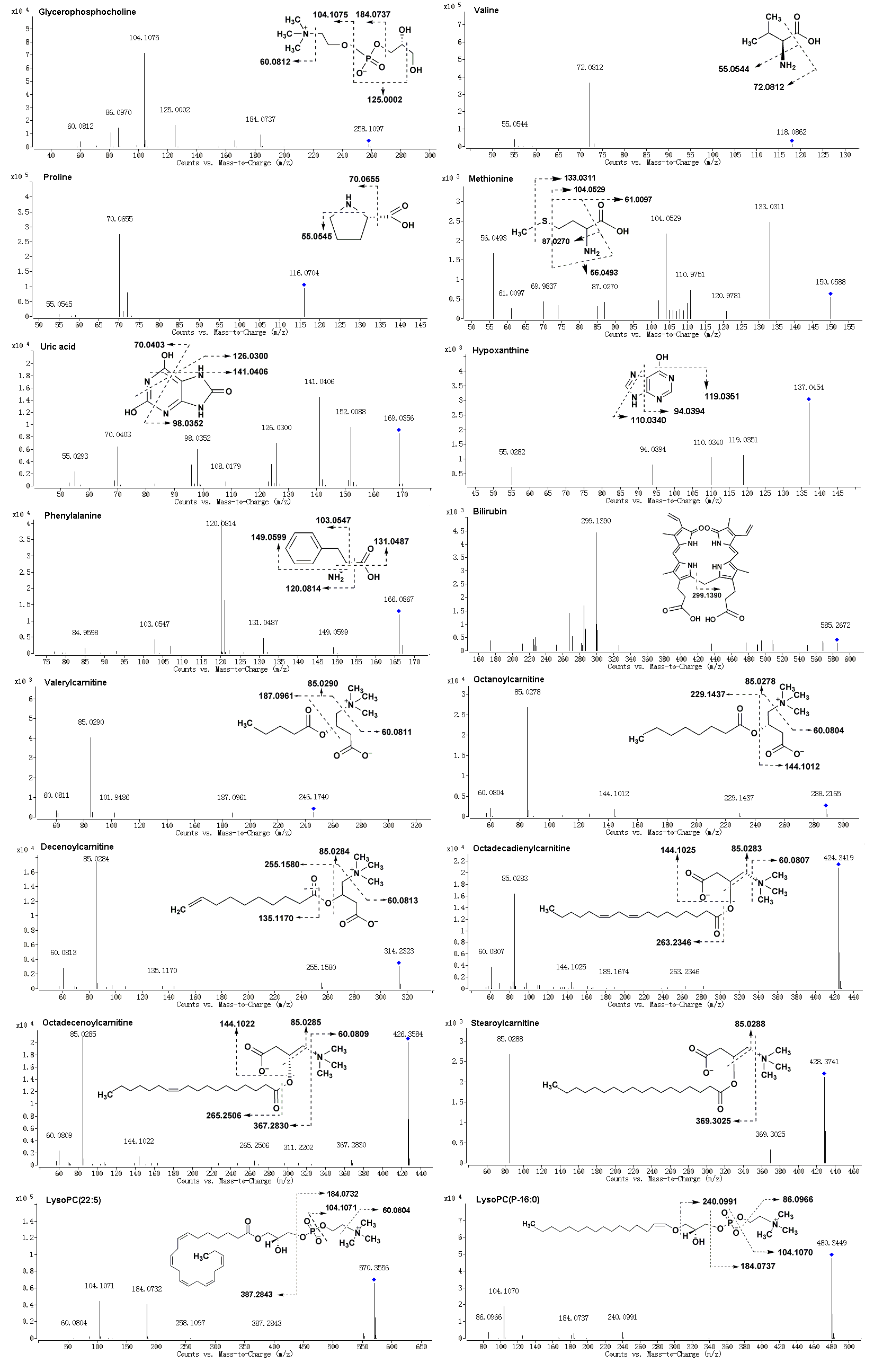


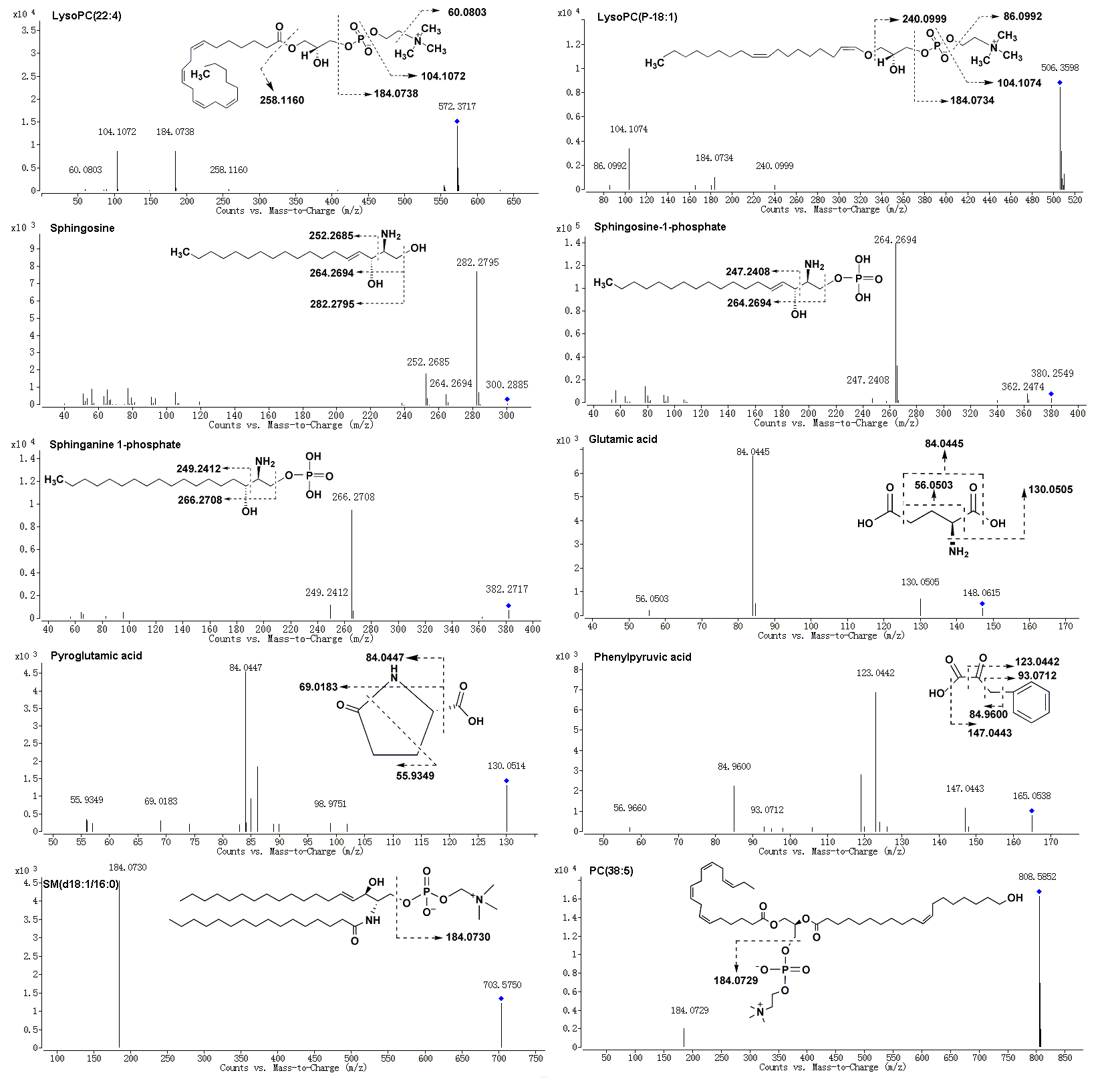


**Figure S6.** Structures and MS/MS spectra of representative metabolites based on plasma spectral data of UPLC-QTOFMS positive ion mode.


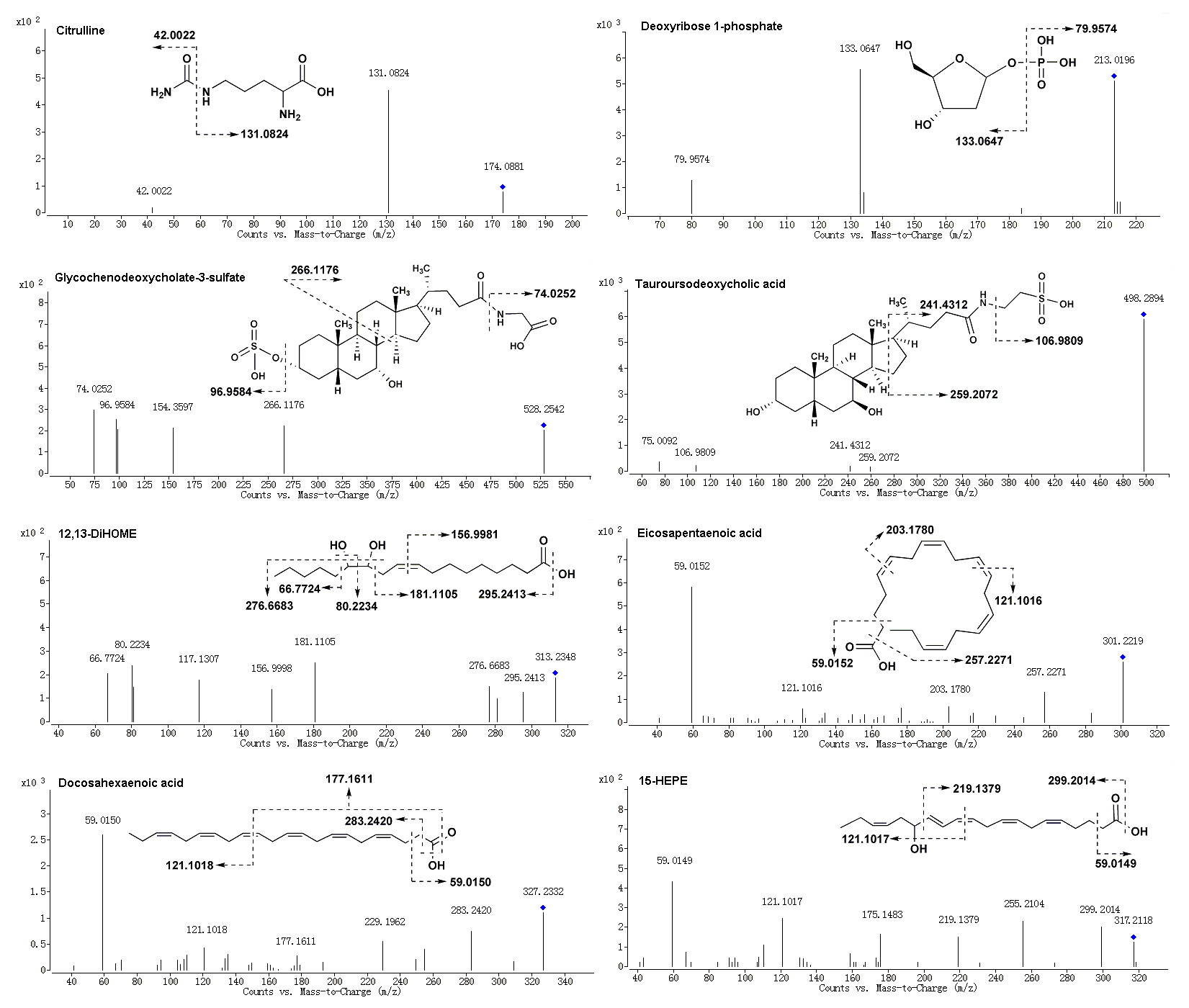


**Figure S7.** Structures and MS/MS spectra of representative metabolites based on plasma spectral data of UPLC-QTOFMS negtive ion mode.

1. Corresponding author: [gaoy66@yahoo.com](mailto:gaoy66@yahoo.com) (Yuqi Gao). [zhanggang196868@163.com](mailto:zhanggang196868@163.com) (Gang Zhang) [↑](#footnote-ref-2)
